# The impact of early intervention psychosis services on hospitalisation experiences: a qualitative study with young people and their carers

**DOI:** 10.1186/s12888-024-05758-4

**Published:** 2024-05-10

**Authors:** Tacita Powell, Nicholas Glozier, Katrina Conn, Rochelle Einboden, Niels Buus, Patrick Caldwell, Alyssa Milton

**Affiliations:** 1 Adolescent Mental Health, Justice Health and Forensic Mental Health Network, Sydney, Australia; 2 Sydney School of Medicine (Central Clinical School), Faculty of Medicine and Health, University of Sydney, 94 Mallett Street, Camperdown, NSW 2050 Australia; 3 The University of Sydney and Australian Research Council (ARC) Centre of Excellence for, Camperdown, Australia; 4 Department of Education, NSW, Sydney, Australia; 5 School of Nursing, University of Ottawa, Ottawa, Canada; 6 Children’s Hospital of Eastern Ontario (CHEO) & CHEO Research Institute, Ottawa, Canada; 7 Susan Wakil School of Nursing and Midwifery, Faculty of Medicine and Health, University of Sydney, Camperdown, Australia; 8 School of Nursing, Western Sydney University, Camperdown, Australia; 9 School of Nursing and Midwifery, Monash University, Melbourne, Australia; 10 Department of Public Health, Aarhus University, Aarhus, Denmark; 11 School of Rural Health, Faculty of Medicine and Health, University of Sydney, Camperdown, Australia

**Keywords:** Early psychosis services, EIPS, Hospitalisation, Mental health, Qualitative, Young people, Families, carers and support people, Australia

## Abstract

**Background:**

While a core aim of early intervention psychosis services (EIPS) is to prevent hospitalisation, many with a first episode of psychosis (FEP) will require inpatient care. We explored young people’s (YP) and their carers’ hospitalisation experiences prior to and during EIPS engagement and how factors across these services influenced these experiences.

**Methods:**

Using purposive sampling, we recruited twenty-seven YP, all of whom had been involved with the hospital system at some stage, and twelve support persons (parents and partners of YP) from state and federally funded EIPS in Australia with different models of care and integration with secondary mental health care. Audio-recorded interviews were conducted face-to-face or via phone. A diverse research team (including lived experience, clinician, and academic researchers) used an inductive thematic analysis process.

**Results:**

Four key themes were identified as influential in shaping participant’s hospital experiences and provide ideas for an approach to care that is improved by the effective coordination of that care, and includes this care being delivered in a trauma informed manner: (1) A two-way street: EIPS affected how participants experienced hospitalisation, and vice versa; (2) It’s about people: the quality and continuity of relationships participants had with staff, in hospital and at their EIPS, was central to their experience; (3) A gradual feeling of agency: participants viewed EIPS as both reducing involuntary care and supporting their self-management; and (4) Care coordination as navigation for the healthcare system: great when it works; frustrating when it breaks down.

**Conclusions:**

Hospitalisation was viewed as a stressful and frequently traumatic event, but a approach to care founded on trust, transparency, and collaboration that is trauma-informed ameliorated this negative experience. Consistent EIPS care coordination was reported as essential in assisting YP and carers navigate the hospital system; conversely, discontinuity in EIPS staff and lack of integration of EIPS with hospital care undermined the positive impact of the EIPS care coordinator during hospitalisation. Care coordinator involvement as a facilitator, information provider, and collaborator in inpatient treatment decisions may improve the usefulness and meaningfulness of hospital interventions.

**Supplementary Information:**

The online version contains supplementary material available at 10.1186/s12888-024-05758-4.

## Introduction

Early Intervention Psychosis Services (EIPS) provide multimodal interventions for young people (YP) with first episode of psychosis (FEP) and, in some programs, YP at ultra-high risk (UHR) of developing psychosis. EIPS aim to reduce the duration of untreated psychosis, improve symptoms, engage the YP in psychosocial recovery and restore a normal developmental trajectory [1]. An additional core goal is to reduce hospitalisation to minimise dislocation of the YP from their community [2] and avoid the potentially traumatic effects of inpatient environments [3]. Meta-analytic evidence suggests that despite there being a lower risk of hospitalisation in the early phase of EIPS treatment compared to treatment as usual, over a third of YP accessing EIPS required inpatient admission [4]. 

Australia has two parallel systems delivering EIPS care. State-funded EIPS teams are co-located with community mental health services within certain local health districts (LHDs) with links to psychiatric hospitals. In addition, a federal government initiative funded six ‘hub and spoke’ *headspace* centres nationwide to deliver EIPS at 14 sites (with a commitment to expanding this to 16 sites announced in 2022). Both state- and federally-funded EIPS aim to deliver care in line with Australia’s national guidelines on early psychosis within the limits of funding, resources, and staffing [2]. State EIPS exist within established LHD health systems, while federally-funded EIPS sit outside these systems [5]. 

For YP, hospitalisation involves risks of disruption to education, peer and family bonds, psychosocial growth, and identity formation during a critical time of development [6]. Hospitalisation during the early course of psychosis has complex effects on a YP’s capacity for self-management and self-determination, impacting appraisals of treatment, illness, and services [7]. Hospitalisation has been found to be a significant risk factor for developing post-traumatic stress disorder (PTSD) following FEP, with one study demonstrating inpatient subgroup prevalence was twice that of the community subgroup [8]. Prevalence rates for PTSD resulting from the trauma of psychotic symptoms and/or hospitalisation range from 11 to 67% with a median rate of 37% [3]. Experiences of seclusion and restraint in Australian hospitals have been described as traumatic, dehumanizing, and “anti-recovery”, where lack of empathy, poor communication and paternalistic attitudes are particularly harmful [9]. 

In the adult mental health literature, a systematic review of qualitative research examining hospitalisation experiences demonstrates the importance of relationships, reducing coercion, safety, and genuine patient-centred care [10]. These findings are mirrored in the literature on YP with FEP, which place a particular emphasis on the negative impact of lack of explanation, confusion, loss of autonomy and powerlessness in hospitalisation [11–13]. The experience of ambivalent feelings toward hospitalisation is a common theme across studies, with hospitals described as places of containment offering both safety from the outside world and a lack of safety due to coercion [10, 11, 13, 14]. Persistent memories of negative experiences are common with protracted involuntary hospitalisation, distressing medication side effects, forced treatment and exposure to violence noted to be traumatising [15]. 

Qualitative studies examining carer experiences of hospitalisation when supporting a YP experiencing FEP have highlighted the distress associated with not receiving help until crisis point, lack of information provision, and the experience of feeling marginalised and distanced from their relationship with the YP [16–18]. Like YP, carers in these studies describe ambivalence, with admission perceived to provide protection as well as harm [17]. 

In general, the literature has focussed on the effect of hospitalisation on engagement with EIPS programs [19, 20]. Coercive hospitalisation experiences, while leading to distress, were not found to impact initial engagement with EIPS [21]. Therapeutic dialogue within a trusting long-term partnership offers a different experience of care compared to hospitalisation, distinguishing it from past negative pathways into EIPS [21, 22]. Conversely, inadequate discharge planning, gaps in service provision and geographical distance of EIPS from the hospital could negatively impact engagement [23]. No qualitative studies have examined the impact of EIPS on further hospitalisations or whether engagement with EIPS prior to hospitalisation impacts negative processes of confusion, trauma, involuntary admission, and length of stay.

Overall, qualitative research has chiefly focussed on the experiences of YP and carers in EIPS programs and how they relate to the recovery process [24, 25]. There remains a gap in the literature regarding the specific effects of EIPS programs on hospitalisation experiences. The aim of this study was to increase understanding of YP and carer experiences of hospitalisation both prior to and during EIPS engagement and explore whether any hospital or EIPS service-related factors influenced these experiences. By comparing experiences of participants from state-based EIPS and federally-funded EIPS we have the opportunity to better understand how systems may influence transitions and collaboration between EIPS and hospitals and how this might impact relationships and effective communication — all of which are key factors in improving hospital experiences in FEP [11, 17]. 

## Methods

### Design

The design was 1:1 semi-structured interviews, analysed thematically [26, 27]. The qualitative semi-structured interview schedule was developed by the research team, including mental health clinicians and researchers (including a researcher with a lived experience of accessing an EIPS in Australia as a client, and a SP) from a diverse range of demographic backgrounds (cultural, gender, region). The study took a critical realist orientation [28] in line with similar research in mental health settings [29] as understanding is constructed from our perspectives and experiences, through what is observable from these interviews. The research drew on qualitative interviews that were conducted as part of the Early Psychosis Youth Services (EPYS) Evaluation project [30], an independent evaluation of the 6 federally-funded EIPS, commissioned by the Federal Government with partners EY (Pty Ltd), the University of Sydney, and The George Institute for Global Health. Full study methods are described in detail in a linked paper [5]. Acknowledging its challenges [31], we made use of the consolidated criteria for reporting qualitative research (COREQ) [32]. See Supplementary File 1.

### Setting

The study took place in six community-based EIPS located in New South Wales and the Northern Territory, Australia. It included services from three new federally-funded services (two in Western Sydney and one in Darwin) and three state-funded services (one in Western Sydney whose catchment overlapped with federally-funded services; two fully integrated with co-located secondary mental health services in inner Sydney).

### Participants, recruitment, and consent

Participants were YP and their carers accessing a participating EIPS. The eligibility criteria for young people included: (1) aged 12–26 years; (2) clinician nominated; (3) minimum two weeks of service engagement; (4) provided written consent (noting additional parent or guardian consent requirements for those under 18 years of age [5]); and (5) an experience with the hospital system. Eligibility criteria for family or carers included: (1) being 18 years of age or over; and (2) being a parent, guardian, family member, partner or friend of a current EIPS client.

Recruitment and the two-stage consent is described in detail elsewhere [5]. In brief, eligible participants were recruited through clinician referral using a purposive sampling approach, in which the study team liaised continuously with recruiting sites to facilitate the recruitment of clients from a range of different clinical stages, ages, genders, and backgrounds including young people from culturally and linguistically diverse backgrounds and those who identified as First Nations people (a person with Aboriginal and/or Torres Strait Islander heritage) [26]. Informed consent was obtained from legal guardian(s) of minor participants following the two-stage process [5]. Only one YP decided not to participate after an initial clinician referral. The number of participants who declined at clinician invitation was not recorded.

### Data collection

After informed consent, audio-recorded interviews were conducted either face-to-face at the EIPS or via telephone between Dec 2019 and May 2020. They were conducted by either AM, a psychologist and qualitative researcher, or TP, a senior psychiatry registrar. A support person was present for the interview if requested by the participant. An interview guide is provided in Supplementary Files 2 and 3. The mean interview duration was 57 min (s.d.: 18 min) for YP and 68 min (s.d.: 17 min) for carers. Participants were provided with a $25 AUD supermarket voucher in acknowledgment of their time.

### Analysis

Interviews were transcribed using the NVivo Transcription Service, then anonymised and checked for accuracy by four members of the research team. Interviews were transcribed as they were conducted, and the team met weekly throughout the interview process to engage in iterative discussion about the main themes. Data were interpreted thematically using an established six step process of qualitative analysis [27] The six steps include [5]: (1) *Become familiar with the data*: the team members were very familiar with the data as they had checked the transcription data for accuracy and engaged in weekly discussions about the themes, and also analysed the data separately in the initial EPYS evaluation (which used top-down framework [impact, satisfaction and culture, and system] to organise inductive findings. Noting that the research reported here was a separate purely bottom-up inductive analysis); (2) *Generate initial codes*: the research group initially explored a sub-sample of data by making comments in the participants own words in Microsoft word document of the de-identified transcripts to develop a preliminary coding framework; (3) *Search for themes*: Open coding was conducted using NVivo 12 Software by one of the analysis team members); *Review and define themes*: the themes in the coding framework continued to be collaboratively refined and named through an iterative process of reading, coding, reflection and discussion in the weekly team meeting until all significant parts of the data had been considered and a codebook was collaboratively developed which included sub-themes and overarching themes. All interviews were subsequently double coded. The collaborative approach to analysis supported reflexivity as it encouraged comparisons and sharing of diverse perspectives the research group offered with their various backgrounds and lived experience– noting overall agreement within the team was high in this study; and, 6) *Write-up*: the results were written up and reviewed by all team members. A lay-summary of findings were returned to participants, however, there was no formal opportunity for participants to feedback on the findings and recommendations other than contacting the researchers directly.

## Results

### Participant hospitalisation background

In total, 27 YP and 12 carers took part in the study. Two YP requested a carer be present who also participated in the interviews. Nearly all (11 of 12) carers were supporting a YP who was interviewed. Demographics of young people who participated in the study are presented in Table 1.

**Table 1:** Participant demographics - young people (*N* = 27)

	n	%
*Months in program (median: 15 months, range: 1–55 months)*		
0–14 months	12	44
15 + months	15	56
*EIPS service type*		
State Funded	8	30
Federally Funded	19	70
*EIPS location*		
Inner Sydney	7	26
Western Sydney	15	56
Darwin	5	19
*Age at time of interview*		
15–19 years	9	33
20–22 years	9	33
23–27 years	9	32
*Gender*		
Male	15	56
Female	12	44
Other	0	0
*Living situation*		
Family	19	70
Partner/husband/wife	2	7
Friend(s)	2	7
State-appointed carer	1	4
Own	1	4
Not specified	2	7
*Aboriginal or Torres Strait Islander*		
Yes	3	11
No	24	89
Not specified	0	0
*Language other than English*		
Yes	9	33
No	18	67
*Education, vocational training or employment*		
Yes	13	48
No	6	22
Not specified	0	0
*Currently in education (including vocational training)*		
Yes	17	63
No	10	37
*Currently in employment*		
Yes	10	37
No	17	63
*Clinical diagnosis*		
At-Risk	5	19
First Episode Psychosis	22	81

All participants experienced an interaction with the public hospital system, via either voluntary or involuntary admission. Only two YP were additionally admitted to a private hospital facility. Two YP reported previously or currently accessing a clozapine clinic run out of the public hospital. The number of contacts with hospital varied, with over half of young people having multiple inpatient admissions, approximately a quarter having one admission only, and a small number presenting to the hospital Emergency Department without an overnight admission. Hospital admission length ranged from overnight to six months. Per standard practice [2, 33], all participants had been assigned a care coordinator at their EIPS, and this individual generally had a clinical or allied health background (for example, nurse, social worker, or psychologist).

### Overarching findings

EIPS intervention positively influenced participant hospitalisation experiences through the care coordinator’s formation of trusting relationships with the YP and family, explanation of why hospitalisation was needed, assistance navigating the system, and advocacy for relevant treatment tailored to the YP. Without this intervention, hospital and community care was perceived to be not only unhelpful but emotionally unsafe for YP and their families. Crises and unexpected events frequently occurred on the recovery journey, and participants highlighted the importance of high-quality, consistent relationships between their EIPS, themselves, and hospital care systems.

Four key EIPS care coordination themes were identified as influential in shaping participant’s hospital experiences and provide ideas to improve care: (1) A two-way street: EIPS affected how participants experienced hospitalisation, and vice versa; (2) It’s about people: the quality and continuity of relationships participants had with staff, in hospital and at their EIPS, was central to their experience; (3) A gradual feeling of agency: participants viewed EIPS as both reducing involuntary care and supporting their self-management; and (4) Care coordination as navigation for the healthcare system: great when it works; frustrating when it breaks down.

### A two-way street: EIPS affected how participants experienced hospitalisation, and vice versa

#### The interplay between EIPS and early hospitalisation experiences

Over half of YP entered an EIPS program after hospitalisation. A small number of YP described their first hospitalisation positively: a refuge providing a break from life stressors. These clients were already linked with an EIPS prior to hospitalisation, and had consented to their admission. For most YP their first hospitalisation experiences were without EIPS support, and they described these negatively. They predominately attributed this to the absence of adequate preparation for hospitalisation, intensifying feelings of confusion.

Over half of YP perceived they were ‘*in prison*’ (P4) during hospitalisations as they experienced lack of activity and stimulation, loss of freedom that was accompanied by surveillance by uniformed staff, confinement in locked spaces, and isolation from their networks and key supporters, with, they felt, little or no explanation. YP described feeling incarcerated for a crime they didn’t understand.

*“Well, I think it’s really scary to be in a place where there’s all these uniforms and it creates kind of a sense of urgency (…) like you’re in prison and you’ve got prison guards kind of thing.”*^*P4*^.

Many carers described having struggled, prior to receiving EIPS support, to integrate dissonant views on hospitalisation; and experiencing tension between relief the YP was getting assistance and fear of potential negative effects. This included fear of rupturing family relationships by opposing YP’s wishes and supporting the admission. A few carers emphasised that they distrusted the approaches to care taking within the hospital setting, and were concerned the YP may learn new unhealthy behaviours or be traumatised by the approaches to care or by other unwell patients.

Many YP not linked with EIPS when presenting to hospital reported entering a high dependency unit (HDU). YP reported this had a considerable impact on them personally. Lower acuity units were preferred compared to HDUs, as admission to the latter was consistently reported to have led to exposure to violence, self-injury, lack of privacy, seclusion, and poor levels of staffing or lack of health care professional expertise. Participants described limited choice over where they (or, for carers, the YP they supported) ended up hospitalised without EIPS.

*“[HDU unit] is mostly used for triage, especially because they are in an area with lots of ice addicts and potential for violence (…) There was a different tone to it. In the [HDU unit] they were trying to defuse people and send them on their way and at [lower acuity unit of first presentation] they were actually trying to help people.”* ^P5^

Positive hospital experiences were linked to recovery-focussed unit culture and peer connections. YP described valuing hospitalisation with other YP experiencing FEP to share experiences and realise they were not alone in their experience of psychosis. YP relayed that this functioned to reduce hopelessness, provide moments of fun, and develop a deeper understanding of their illness. In contrast, a smaller number of YP expressed a preference for limited engagement with others, as their level of perceived trauma increased when exposed to other’s mental health issues.

*“I personally think that these spaces should not be traumatic, but they are. And unfortunately, I can’t say whether that’s because of the system, because of the kids themselves and their own issues. And it’s just like bouncing off this depressed person’s depression. This person’s got bipolar and schizophrenia and anorexia. And so, like you’re hearing all these stories”* ^P26^

Carers’ attitudes towards the hospital unit mirrored those of YP, with additional importance placed on family inclusivity and consultation, facilities being youth-friendly, and families’ being welcome at the unit.

The small number of YP who were not referred to an EIPS on discharge from hospitalisation spoke of the futility of the admission and the long-lasting impact of social withdrawal. The majority of these YP reported their mental health deteriorated rapidly over 1–2 months into acute relapse.

*“There was no forward motion. I just felt stagnant. And that just made me feel like crap. And that just sent me down a spiral which led to psychotic depression, which got me back into a psych ward.”*^*P5*^.

For two YP, it took until their third admission (over a period of several months) to be referred to an EIPS. Engagement with EIPS broke their cycles of rapid re-admission. The issue for these two participants was a lack of appropriate referral to EIPS from the hospitals involved. One was ultimately referred to a state-funded service; one to a federally-funded service. One other YP reported securing an EIPS place via a family friend working in healthcare, rather than a professional care provider.

#### Reducing readmission to hospital through effective care coordination

Once linked with EIPS, over a third of YP described managing crises or early warning signs of relapse *without hospitalisation*. Avoidance of hospitalisation was in part attributed to the program and their care coordinator. Participants described this occurring through effective detection of early warning signs and adjusting medication, increasing frequency of care, modifying precipitating stressors, safety planning with the YP and carers to set up the home for acute illness management, and hospital staff deciding against admission due to a community option for assertive care. Being visited at home was preferred as it felt familiar and safer.

*“It was kind of scary to go to a hospital because you don’t know what’s going to happen with you there. At least, at home I know I’ll be safe and not, I guess, overthinking too much and stressing out. I’m at home with people that I know, not at a hospital where nurses give you medication and whatnot, to calm you down and being around other unwell people and that.”* ^P9^

For a small number of acutely unwell YP, assertive treatment services (Mobile Assessment and Treatment (MAT) Team in federally-funded EIPS; and the Acute Care Service in state-funded EIPS) were needed, including home visits, until their mental health stabilised. This assertiveness was described as essential in managing the YP’s difficulty attending EIPS appointments during regular hours due to symptoms. One YP described how phone calls to check in were essential during a relapse, especially when symptoms of acute psychosis led to avoidance of services.

*“At the time, it’s not too good because obviously I don’t want to be spoken to, but when they can get a hold of me and I can talk about it, it’s obviously life changing. The matter of me picking up the phone and talking about what’s wrong.”* ^P27^

In contrast, communication delays with EIPS during crises were identified by approximately a third of participants as leading to negative re-hospitalisation experiences. One carer reported attempting to call the MAT team in the federally-funded service, and not receiving a call back until the following day. They felt this had significant consequences for the YP, who by then had been admitted to hospital outside his LHD.

*“If I had been able to get in contact with his MAT team, a psychiatrist might have been able to come out and say, take this, because the antipsychotics work pretty quickly (…) So I do wonder if we had had access to the MAT team at that point, that maybe that whole extra hospital stay wouldn’t have occurred.”* ^P15^

Longer engagement with an EIPS meant YP felt more empowered to articulate symptoms, express and negotiate preferences, and engage in treatment to avoid hospital. Some YP described discussions of the need for acute hospitalisation with their care coordinator as stimulating shifts in awareness and precipitating changes in their self-management skills.

*“[EIPS] was ultimately saying, we don’t want you to go to the hospital. Our whole main aim is to prevent you from going to hospital and to prevent any early psychosis. So, they like embedded that into me. They were like, hospital is not a good thing. You don’t want to go to a hospital, you want to stay at [EIPS] and be able to manage your mental health.”* ^P19^

*“I decided not to [attend hospital] because I thought maybe I can deal with that myself. Maybe I can try doing things myself, because at that time, I wasn’t putting in the strategies. It had only been when I’ve been using my strategies to deal with my psychosis that I had gotten better over a period of time.”* ^P26^

### It’s about people: the quality and continuity of relationships participants had with staff, in hospital and at their EIPS, was central to their experience

Participants appreciated open, honest, and relaxed communication in EIPS staff, and care coordinators in particular held distinct value in their depth of YP knowledge as well as their location outside the hospital, which was perceived as a coercive system. YP highlighted the importance of trust building, working through ambivalence over treatment options, and processing the experience of hospitalisation.

Approximately half of YP described mental illness influencing their perception of hospital and EIPS staff and the meaning they attributed to hospitalisation. Many YP reflected on this experience in retrospect, acknowledging the influence of illness on formation of trust, interpretation of events experienced and engagement in their treatment. YP spoke of being acutely confused, developing paranoid beliefs about staff and other YP that may not have been grounded in reality, and experiencing fear due to stigma and social narratives of psychiatric hospitals.

*“I tried to explain all my fantasies and all I was hearing and seeing. I had psychologists and psychiatrists and they were trying to challenge those thoughts, which is really hard to like kind of comprehend because at that time those thoughts were really strong. That’s probably another reason why I wasn’t as enthusiastic about going to (EIPS). So it’s like the nature of psychosis made it harder*”^P19^.

This made long-term relationships built with consistent care providers in EIPS especially important in navigating the non-linear experience of recovery and working through ambivalence to facilitate engagement.

Over half the interviewed participants reported that staff inconsistency, low skill, and turnover could undermine the development of a therapeutic relationship and the critical role the care coordinator could play during a hospitalisation. Participants reported more access to senior permanent psychiatrists and lower care coordinator turnover in the state-funded EIPS (except in Darwin, where the federally-funded service is the only EIPS in the region, and where there are strong links between this EIPS and local health services).

*“I feel like it depends on your case manager. I actually don’t think that my first case manager when I went to my first hospital admission focused on it well… So, it wasn’t as helpful. But my second and third admission, I had the same case manager. So, it was much easier for me to do the mental health plan. The techniques were thoroughly taught. So, it really depends on the case manager.”* ^P19^

One YP spoke of being involuntarily admitted in the middle of the night to an adult hospital because of unstable staffing arrangements in their federally-funded EIPS.

*“I just felt like I was just one of those people that fell through the cracks sort of thing. I guess… well, like the lack of understanding and well now, because they’ve gone through some changes, and I’ve been through so many different people. I just felt like my case wasn’t really looked at properly.”* ^P13^

The processing of hospitalisation experiences involved understanding how the YP ended up in hospital, how to make sense of what happened to them and how to avoid re-admission.

*“Well, the comparison to [first admission prior to EIP] I didn’t really walk out with an understanding. I just walked out with a lot more questions than answers and a lot more stress. While, when I came back here [after second admission], I could kind of like, ask ‘What happened? Why am I like this?’ I could actually get a response back, and a strategy back on how to prevent this, and how to talk better about this.”* ^P14^

Within the hospital, negative experiences of confusion, trauma, and coercion were frequently reduced when a trauma-informed approach to care, including authentic interest, empathy, consideration of preferences and flexibility, was provided. Authentic communication was described by many YP as listening deeply and treating the YP as a ‘*normal person, not sick.’*^P25^ Nurses and allied health were mentioned as facilitating these experiences of feeling understood, in contrast to frequently disconnected experiences with ward-based doctors.

*“(Hospital doctors) are disengaged from us, like the nurses live with us in that situation (…) it’s the doctors who make the final call, but they’re the ones who know you the least*”^P4^.

*“So I feel like when I was there, it didn’t really get to the root of, you know, they didn’t seem too concerned about what was actually happening. And then like, I got frustrated with them because the psychiatrist that would come in and talk to me, she just annoyed me because she seemed like she knew everything without really talking to anyone.”* ^P12^

A third of YP perceived that a thorough understanding of their case did not appear to be a priority of the hospital. They described a lack of transparency in hospital processes, history taking without contextualisation of their presentation, and limited attempts to understand where the YP was “*coming from.*”^P7^.

Feeling ignored led to YP feeling like they needed to “*play by the rules if I want to get out of here.*”^P4^ This feeling was coupled with YP minimising the impact of their symptoms and performing acts that they thought would be viewed by hospital staff as reflecting good functioning and being well.

*“Because, you will feel confronted and instead of you opening up more, you will just, you know, cover yourself because people are not taking you seriously.”* ^P7^

Carers similarly identified that quality of relationships made a significant difference to the perception the YP was being cared for. Carers described being frustrated by a lack of communication and poor responsivity to requests, which led them to feel that their knowledge and understanding of the YP was being ignored.

*“So, he was in hospital for two months and they contacted me three times, despite me trying to contact them. Well, I was in there every day, I would say, can you please call me so I know where to come for a meeting. They said, ‘we don’t need to see you’. Well, you do. I found their lack of communication staggering.”* ^C8^

Confidentiality limited disclosure of information by hospital staff and EIPS care coordinators. These limitations meant that one in three carers felt excluded from understanding the full story of their YP’s illness and progress.

*“It was pretty hard and it has still been very hard. Because we only know bits and pieces. We don’t know the full issue.”* ^C12^

### A gradual feeling of agency: participants viewed EIPS as both reducing involuntary care and supporting their self-management

YP perceived EIPS engagement impacted the use of involuntary care with no notable differences between state- or federally-funded EIPS. Approximately half of YP were admitted involuntarily for their first admission. Those who experienced further hospitalisations in the EIPS program were more likely to be voluntary for psychosis, and involuntary for overdoses and suicidal ideation. The small number of YP that continued to be admitted involuntarily for psychosis had a higher burden of residual psychotic symptoms and were less engaged and adherent with medications.

Being involuntary was described as aversive for YP due to the involvement of police and ambulance, use of force, coercion to take medication, leave and discharge being denied, a sense of intimidation during the tribunal experience, and the perception that there was no pathway to voice disagreement.

*“Involuntary is horrible. That’s one of the bad things. The first two times in [acute unit] I had to go to tribunal. That was horrible. I don’t think that they should put people through that… It’s like being in a court case or something. And you’re a schizophrenic, thinking they can read my mind and it’s just horrible.”* ^P24^

Some voluntarily-admitted YP perceived a more covert coercion, feeling that their status would change to involuntary if they made choices that were not aligned with the treatment preferences of the hospital team.

*“I stayed voluntarily. So, if I do anything, that’s against what they say to do, so if I choose to not take my medication, you know, it’s essentially you don’t have a choice because you came here voluntarily. So, we’ll switch you to involuntary, which means your stay’s indefinite.”* ^P26^

Some YP described a relationship between coercion and stage of illness and that coercion might diminish as the hospital staff became familiar with them and symptom reduction allowed for dialogue around decision making.

*“It was very coercive, everything. So, when you’re sleeping, we’re going to give you the medication, open your mouth and things like that. You know, it was really, ‘wow, you don’t treat, you don’t treat anyone, anyone like this’, you know what I mean? So that really got a little bit under my skin. (…) But then later when they started trusting me more, they, they were, they started like listening to me and what I wanted to tell them about medications.”* ^P7^

A small number of YP reported feeling in control during a voluntary admission. They relayed feelings of choice and self-responsibility.

*“Being voluntary, just a word, it just makes you feel more like you have more control over yourself. And in involuntary, it’s like jail. You just don’t know when you gonna leave. With voluntary at least, you know you have the power to fix yourself.”* ^P24^

In contrast to coercive hospitalisation experiences, over half of YP described noticing how the EIPS team considered their preferences and emphasised choice. A shift in focus towards self-management and self-responsibility was described by over two thirds of young people, with over a quarter describing it as being the most significant part of the EIPS program.

### Care coordination as navigation for the healthcare system: great when it works; frustrating when it breaks down

Over half of participants reported EIPS advocacy in hospital was beneficial. This facilitated effective communication between them and the hospital, and positively impacted treatments or duration of stay. In contrast, around a third of YP attributed their increased confidence with re-admission to their own personal development (rather than care coordination), familiarity with the hospital, and a greater self-awareness of symptoms including what to say and what not to say to navigate the system to their advantage.

*“This is my full fourth hospitalization. So I’m a lot more use to it, and the doctor goes to me, ‘You sound different this time.’ So I was talking all delusional. But I was just keeping it to myself and just talking normally. So even though I went back to hospital, I was like, ‘Oh, well, I know I’ll get back on my feet’, whereas the first hospitalization, I thought the whole world was disappearing.”* ^P24^

#### Facilitation of planned and unplanned hospitalisation

For approximately a quarter of participants, negative attitudes to hospital shifted when hospitalisation was planned and used purposefully. EIPS aided this process through appropriate hospital choice, and when admissions were utilised to safely manage medication changes. These participants were markedly more likely to also describe the benefits of co-location of an EIPS and hospital within the same LHD, with a shared clinical records system and a clear relationship between the hospital, acute care service and their EIPS.

*“So they have good communication with the EIPS team because you know, it’s literally 200 metre walk from one place to another. It’s not hard to communicate with the other place. They had constant communication, so I think it was very beneficial to both parties to be able to, you know, head back and forth like, you know, what they think that [name] should be doing, and whether or not she should be leaving, I think it gave both parties confidence as well.”* ^C2^

One hospital in NSW was described consistently as having a strong relationship with a federally-funded EIPS. This improved the relevance of intervention offered, integrated points of care, and improved handover of information to prevent repetition.

When the EIPS was in a different LHD than the admitting hospital, communication broke down with care coordinators not being informed of admission. The persistence of care coordinators in advocating for shared discussions was valued.

*“One day, [case manager] actually insisted that we’ll have a meeting together. And we were in the meeting and she was asking questions. And if it wasn’t for her, I don’t believe we would have gotten anywhere.”* ^C8^

For most participants, when the care coordinator was unable to take an active role in the admission this had negative consequences for YP’s experiences. One YP spoke of the difficulties of not being visited by her care coordinator during her hospitalisation where she was physically assaulted. She felt communication would have informed the hospital of her vulnerabilities and advocated for better management of unit dynamics, which she was too afraid to discuss with hospital staff.

*“I think it would have been really reaffirming like ‘I’m still here for you’. You know, like ‘You’re still my client, I’m still going to help you. And at the end of the day, this is for you to help you’”* ^P26^

A few YP spoke of the negative effects of being prescribed medications without EIPS consultation resulting in significant side effects. A few young people raised how lack of understanding of their case could have been avoided if EIPS were leading the admission process.

*“If (EIPS) had their own mental ward, I would admit myself into that and it’d be sweet.”* ^P27^

A small number of young males reported not wanting care coordinator collaboration in hospital. These YP presented either with a higher symptom burden or a desire for control of information and choice over EIPS involvement. They declined to let the EIPS team know when they were being admitted, perceiving the team would have had no impact on the admission process and their care.

#### Length of hospitalisation

Over half of participants perceived being part of an EIPS program influenced length of hospitalisation through collaboration of the care coordinator and the hospital. When the YP had only recently entered the program prior to first hospitalisation this effect on length of hospitalisation was lost.

*“Not necessarily, just because of how close to the end of the bridge I was when I first came into [EIPS] I feel like if I knew how to actually get in contact and make an arrangement sooner, I could have avoided hospital altogether.”* ^P12^

Approximately a quarter of YP commented there appeared to be no connection between being part of the program and the length of their hospitalisation. This was linked to admissions with a clear demarcation of roles between the EIPS and hospital, with decision-making responsibility lying solely with the hospital psychiatrist.

Conversely, a few participants commented the advocacy of their care coordinator lengthened hospital stay appropriately to address unresolved psychotic symptoms and allow time to address psychosocial factors.

## Discussion

### Principal findings

This is the first Australian qualitative study to explore YP and their carers’ hospitalisation experiences both prior to and during engagement with EIPS. Inclusion of YP at different illness stages and time in an EIPS aids understanding of how experiences contrast at different stages of recovery and engagement. Participants consistently described EIPS and hospital as contrasting experiences of relationships, coercion, and transparency. Many participants noted hospital communication could be improved, with some participants describing formative moments of care and connection with specific hospital staff members. This mirrors research demonstrating compassionate relationships modify negative experiences of inpatient loss of autonomy [34]. These findings point to a need for integrating structures of support to provide a sense of safety to YP and their families in navigating hospital systems.

### The impact of health service design

The importance of coordination between EIPS, the associated acute after-hours service, and the local hospital was highlighted. Being an EIPS client was not sufficient to avoid hospitalisation, but service and funding structures could support an integrated approach to care, with significant effects on YP outcomes. Service co-location, shared information systems and inter-service case conferences were valued because a care coordinator working for an EIPS within the same information system as the hospital could offer relevant context to support tailoring interventions in a meaningful way. They clarify processes and rationales for approaches to care, and support a transfer of trust and knowledge. Additionally, YP and their carers indicated EIPS shaped their perceptions of hospital admission overall, by minimising urgent, unexpected admissions on the one hand and facilitating (and increasing the perceived utility) of essential admissions on the other. They criticised what they perceived to be hospital structures that placed decision-making power in the hands of psychiatrists with whom they had no previous relationship from which to build trust or familiarity.

Internationally, embedding of EIPS services within healthcare systems has faced difficulties, with lack of collaboration between clinical infrastructures, access inequality, and service incapacity due to insecure staffing [35]. Study participants at federally-funded EIPS experienced greater difficulties with staff continuity than state-funded services, which clearly needs to be addressed.

In line with past research [36], participants generally preferred home-based treatment. Alternative models to inpatient treatment offer less restrictive care and choice, key themes identified as important. Reviews of alternative acute care models in the UK highlight the complexity of diversity in services, as the benefits of increased choice need to be weighed against loss of continuity and confusing system pathways [37]. Our study illustrates the importance of improving continuity and coordination between EIPS and acute care services and hospitals, and for the federally-funded teams to develop improved relationships with the public health system.

Hospitalisation punctuates FEP and the recovery process, forming a critical juncture where hospital care impacts YP pathways in illness management and personal transitions including the integration of illness into identity. An active and collaborative care coordinator was portrayed as the thread that connected different crises and hospital experiences, creating a line of connection that enabled participants to feel supported. The intensity, length and subsequent trauma of hospitalisation experiences were reduced by preparation done with participants, liaison with hospital teams and processing post-hospitalisation. Practical interventions included calling ambulances, informing family, providing handover to hospitals, and supervising leave. Holistic interventions included promoting reflection on symptoms, beliefs, and perceptions to process and make meaning, increasing the usefulness of a hospitalisation event. Processing hospitalisation and self-management interventions were described as profound by many participants at a later stage of recovery, compared to those in early recovery where practical supports were most valued. The importance of post hospital support has been detailed in our linked paper focussing on service transitions, which found intensified follow-along support from EIPS was critical with a need for consistent, coordinated discharge planning with carer and YP involvement.5 The work of carers and care coordinators has been reported in a meta-synthesis as essential in integrating hospitalisation into larger narratives of recovery and self-management by restoring a sense of control and agency [24]. Our study supports previous findings from FEP engagement literature that time, stage of illness, and individual experience of recovery influence the care coordinator/YP relationship [38]. Previous authors have described the importance of relationships as a dynamic bidirectional process [20]. In our study, the presence or absence of a consistent care coordinator who had been able to build this relationship with their client, as well as the individual characteristics of YP and their carers, combined to create quite varied hospitalisation experiences across our participant group.

### Impact of family systems

This study was unique in integrating hospitalisation experiences of both YP and their carers. Previous research has highlighted interactions between the initial carer-YP relationship, carer appraisal of YP illness and behaviour, carer experiences with services and subsequent carer behaviour towards the YP [16, 39]. Many YP and carer narratives converged in raising similar themes with key differences in perspective due to their distinct vantage points being accommodated within or outside the hospital system. A few YP and carer perspectives diverged which was linked to the nature of their relationship and differing perceptions of illness. Carers spoke of EIPS advocacy needed to obtain information in hospital. The care coordinator was pivotal in contextualising the illness, providing education on the purpose of hospital, and assisting with modification of negative carer attitudes linked to social stigma and cultural beliefs. There was a continuum of carer involvement in YP recovery processes, and this influenced the extent to which the carer or the care coordinator took the lead in driving collaboration in hospital. In cases where YP restricted the flow of information to their carer for confidentiality, the care coordinator played a critical role in managing YP and carer experiences. The federally funded EPYS addressed family dynamics influencing YP hospitalisation experiences through service family therapy interventions and family peer support recognising reciprocal interactions between family relationships and stress and carer burnout and YP disengagement. Family interventions in FEP is supported in the literature with evidence carers can reduce rates of relapse and hospitalisation [40] and facilitate acceptance of illness and treatment [21]. Participant experiences mirrored findings in the literature of the importance of information sharing, carer involvement in care planning and discharge and valuing carer knowledge [41]. Our findings suggest greater integration of family support into hospitals is needed. This is supported in the literature which shows the need to include family support as core business for inpatient staff and for early contact and partnership in decision making [42]. 

### Reducing coercive practices and promoting trauma-informed care

Unsupported admissions (i.e., those without a care coordinator) were frequently traumatic for YP. This finding resonates with prior research where the absence of information about what to expect in hospital and what was happening during admission intensified fear and confusion [11]. Our findings suggest that, where possible, engaging participants prior to escalation provided greater opportunity for mitigating potential for trauma from hospitalisation. Tailoring care to the context of the YP and family, and preserving their agency within decision-making by offering an option for treatment within the home environment, created a sense of safety [43]. Similarly, the formation of long term relationships with care coordinators privileging self-management and personal recovery goals diminished participant powerlessness over the hospitalisation process. This approach aligns with a Trauma-Informed Care (TIC) perspective, which appreciates the importance of recognising and addressing powerlessness of YP and families and offering real choices in the context of inpatient psychiatric care and involuntary care legislation. Reducing iatrogenic trauma has the potential to modify the recovery course, due to the dynamic interplay between traumatic stress and psychosis. YP valued receiving assistance to process hospital related trauma at their own pace by their EIPS care coordinator, mirroring prior research [44, 45]. 

Participant reflections highlight the complexity of involuntary treatment. YP reported ambivalent emotions over hospitalisation experiences, with some YP recognising the relationship between experiencing hospitalisation as frightening or prison-like and the impact of FEP on self-awareness and their decision making, acknowledging why involuntary interventions had been used in the context of psychosis. A review of randomized studies revealed that increasing shared decision making and hospital staff training in least restrictive practice reduced coercive treatment [46]. The potential use of advanced statements in FEP has been explored in a qualitative study which identified the benefit of the tool in empowerment, prompting discussion of client preferences, and communication of YP information to optimise treatment [47]. Use of this instrument could modify the impact of involuntary treatment by providing a plan for treatment previously consented to by the YP improving the relevance and acceptability of treatments offered in hospital. The statement could potentially modify the risk of traumatic experiences by detailing the YP’s trauma triggers and personal preferences for care.

### Strengths and limitations

Key strengths of this study include that we were able to capture the perspectives of a large and diverse group of YP and their carers, across multiple service locations, due to a purposive sampling strategy that was largely successful. As it was part of a larger evaluation, we were not able to assess data saturation and we relied on a priori estimates of an appropriate sample size based on literature guidance [48, 49]. General limitations included recruitment through EIPS clinician nomination increasing the possibility of gatekeeper bias, sole reliance on participant recollection, and limitation to the Australian, chiefly urban, context.

Specific limitations include not exploring the influence of culture, ethnicity, or socioeconomic structural adversity on participant hospitalisation experiences. Further research into the intersection of these factors with EIPS support is required as Australian born migrants with FEP have longer admissions and high rates of involuntary care [50]. 

Although our study highlighted the importance of earlier engagement and improving accessibility of EIPS, further research is needed into what subgroups of YP presenting with FEP are more likely to be hospitalised. The drivers of hospitalisation are complex, and previous research highlights the importance of addressing clinical and system factors. It is possible those linked with EIPS prior to hospitalisation represent a subgroup with specific clinical and socioeconomic characteristics.

## Conclusions

The positive impact of EIPS on hospitalisation was frequently associated with a trusting relationship with a consistent care coordinator, who demonstrated expertise in tailoring an approach to care through early engagement with the YP when experiencing increasing psychosis symptoms, a non-coercive practice approach, and partnership with hospital care providers. Discontinuity in EIPS staff and lack of integration with hospital systems undermined the care coordinator’s role in hospitalisation. Information provision and developing YP recovery growth appeared to increase participant confidence in hospitalisation processes. Family work with carers was valued. Care coordinator involvement as partners in inpatient treatment decisions may improve the usefulness and meaningfulness of hospitalisations.

### Electronic supplementary material

Below is the link to the electronic supplementary material.

**Supplementary Material 1**: COREQ Checklist

**Supplementary Material 2**: Young Person Interview Guide

**Supplementary Material 3**: Support Person Interview Guide

## Data Availability

Due to the conditions outlined in the study’s consent process, full transcripts cannot be shared to protect participant anonymity. However, amalgamated datasets generated and/or analysed during the current study are available from the corresponding author on reasonable request.

## Declarations

### Author information

AM (BSc, PGDip Psych, MAppSc Health Psych, PhD): is a psychologist and research fellow with Australian and international experience working with EIPS and adult mental health services. TP (BSc, MBBS, M Psychiatry, M Clinical Family Therapy) is a child and adolescent psychiatrist with experience working in child, youth (EIPS) and adult mental health services. KC (BSc, Bed) is a teacher and lived experience researcher with a lived experience of using EIPS. RE (BScN, MN, PhD) is a nurse and qualitative researcher specializing in critical social theory. NB BN, MScN, PhD) is a mental health nurse and professor in qualitative research and qualitative research methodology, with a focus on critical health research, ethnographic theories and methods. PC is a medical student. NG (MA, MBBS, MSc, MRCPsych, FRANZCP, PhD) is a consultant psychiatrist specialising in epidemiology, trials and health services research.

## Abbreviations

EIPS: Early Intervention Psychosis Services
EPYS: Early Psychosis Youth Service
FEP: First episode of psychosis
UHR: Ultra-high risk
