# Supplementary material for: The impact of early intervention psychosis services on hospitalisation experiences: a qualitative study with young people and their carers
Source: BMC Psychiatry. 2024 May 10;24:350. doi: 10.1186/s12888-024-05758-4 (PMC11088060; doi:10.1186/s12888-024-05758-4)
Supplement: Supplementary file 1 — Supplementary Material 1: COREQ Checklist [file 12888_2024_5758_MOESM1_ESM.docx]

**Supplementary File 1.** COREQ checklist

Authors: 1. Tacita Powell, 2. Nick Glozier, 3. Katrina Conn, 4. Rochelle Einboden, 5. Niels Buus, 6. Patrick Caldwell, 7. Alyssa Milton

| **Number** | **Item** | **Description** | **Page Number** |
| --- | --- | --- | --- |
| 1. | Interviewer | Authors 1 and 7 conducted the interviews with young people and their carers accessing EIP services (state and federal). | 8 |
| 2. | Researcher credentials | Author 1: BSc, MBBS, M Psychiatry, FRANZCP  Author 2: MA, MBBS, MSc, MRCPsych, FRANZCP, PhD  Author 3: BSc, BEd  Author 4: BScN, MN, PhD  Author 5: BN, MScN, PhD  Author 6: BA, LLB, MPH, GradCertClinEpi  Author 7: BSc, PGDip Psych, MAppSc Health Psych, PhD | 18 |
| 3. | Occupation | Author 1 is a consultant psychiatrist who has experience working with EIPS services  Author 2 is an academic psychiatrist.  Author 3 is a teacher and lived experience researcher  Author 4 is a nurse and qualitative researcher specializing in critical social theory.  Author 5 is a mental health nurse and specialist in qualitative research and qualitative research methodology, with a focus on critical health research, ethnographic theories and methods.  Author 6 is a medical student with experience in qualitative and quantitative research  Author 7 is a psychologist and research fellow specializing in qualitative and mixed methods research and has Australian and international experience working with EIPS. | 1 |
| 4. | Gender | Authors 2, 5, and 6 are male.  Authors 1, 3, 4, and 7 are female. | 1 |
| 5. | Experience and training | Authors are experienced and active researchers with expertise in qualitative, quantitative, and mixed methods approaches. Authors have researched and/or published in the broad topic area previously. | 1, 7 |
| 6. | Relationship established | A two-stage consent process was applied, where clinicians briefly described the study to potential participants meeting eligibility criteria gaining consent to refer to the researcher. The clinician scheduled the interview for those expressing interest in participating. Prior to interview, participants had the opportunity to review the participant information and consent forms, and discuss any questions, before giving informed consent. Participants aged 12-18 years required parental/guardian co-consent, with 16-18 year olds parental/guardian consent being subject to clinician advice and state specific laws. | 7, 8 |
| 7. | Participants’ knowledge of the interviewer | All interviewers had no previous professional or personal relationship with any of the participants. Co-authors only reviewed de-identified data thus had no personal relationship with, or knowledge of participants. Participants were informed about who the involved researchers were in the Participant Information Statement (PIS). | 8 |
| 8. | Interviewer characteristics | The interviews were conducted by a psychologist and researcher with experience in qualitative and mixed-methods research in Australian and international mental health services including EIPS (Author 7) and a consultant psychiatrist with experience in community-based EIPS, pediatric and adult mental health services and hospital services (Author 1). Interviews were supported by the research team which included Author 3 who is a researcher with lived experience of using EIPS as a client. | 7, 8 |
| 9. | Methodological orientation and theory | Thematic analysis with an inductive approach was applied, using a critical realist orientation. | 7, 8 |
| 10. | Sampling | To enable participation in the research, clinicians were asked to nominate and invite all clients (and their SPs) on their caseload who met eligibility criteria and had capacity to consent. Purposive sampling was used to recruit a diverse sample of the EIPS client population and their support people (family, partners or carers) who had an experience with the hospital system either personally or via the young person they support. Representation from special interest groups was prioritised. Special interest group of clients and family / carers represented various clinical stages (UHR, FEP), ages, genders, culturally and linguistically diverse and Aboriginal or Torres Strait Islander backgrounds. Sampling did not require researcher access to client records. The researchers fed back to the coordinating clinicians at each EIPs if there were any recruitment gaps and clinicians checked their entire caseload for eligibility so as to minimise the potential for gatekeeping and bias. | 7, 8 |
| 11. | Method of approach | Eligible participants were recruited through clinician referral. EIPS clinicians and managers spoke of the study to potential participants and sent communications to clients and support people advising of the study. Clinicians invited all eligible young people on their caseload who met criteria and had capacity to consent. | 6, 7 |
| 12. | Sample size | Semi-structured interviews were conducted with 27 young people accessing EIPS, and 12 carers. | 7, 8 |
| 13. | Non-participation | One young person chose not partake in the interview after reading the participant information statement. All interviews that commenced were completed. The number of participants who declined at clinician invitation was not recorded. | 8 |
| 14. | Setting of data collection | Interviews were conducted face-to-face on the EIPS premises or via telephone between Dec 2019 and May 2020. | 8 |
| 15. | Presence of non-participants | Participants could choose to have a support person or advocate present at the interview (who could also consent and contribute to the interview), however this was not required for any interviews. Two YP requested a support person to be present who also participated in the interviews. | 9 |
| 16. | Description of sample | A diverse sample of young people and their support people (parents or partners) receiving support from Australian state or federally funded EIPS.  Participants were YP and their SP accessing a participating EIPS. The eligibility criteria for young people included: (1) aged 12-25 years; (2) clinician nominated; (3) minimum two week service engagement; (4) provided parent or guardian if aged between 12-15 years, and when advised by the clinician, aged between 16-18 years. The eligibility criteria for family or carers included: (1) being 18 years of age or over; and (2) being a parent, guardian, family member or friend of a current EIPS client. | 6-8, Table 1. |
| 17. | Interview guide | Interviews were semi-structured. Questions covered: Client experience of coming into the program; Client experience of the program; Client views on the impact of the program on their functional outcomes; Client hospitalisation experience whilst involved in the program; Treatment (medication, CBT, family care); Ongoing community care, mobile outreach and group programs; Family programs and family peer support; Youth participation and peer support program. In order to enhance question relevance, a lived experience researcher who had used EIPS contributed to the interview design. | 7, 8, Supplementary File 2. |
| 18. | Repeat interviews | N/A | N/A |
| 19. | Audio/visual recording | Interviews were audio-recorded. | 8 |
| 20. | Field notes | NA | NA |
| 21. | Duration | The average interview duration was 57 minutes for YP and 68 minutes for carers. | 8 |
| 22. | Data saturation | Ensuring sufficient numbers of participants were recruited for the purposes of theme saturation was guided by Hagaman and Wutich (2017) and Hennick et al. (2017) indicating 20-40 participants would be required. This saturation guidance was selected as the research involved recruiting a non-homogenous participants (ie. both YP and SP) and was run across multiple EIPS settings across Australia with different funding (state and federal). As it was part of a larger evaluation, we were not able to assess data saturation via a stopping criterion, however, we as described above relied on a priori estimates of an appropriate sample size to reach saturation. | 9, Table 1. |
| 23. | Transcripts returned | Transcripts were not returned to participants. A lay-summary of findings was returned to participants. | 8. |
| 24. | Number of data coders | Data were coded by author 1 and supported by authors 6 and 7. | 8 |
| 25. | Description of the coding tree | Codes, themes, and subthemes were iteratively refined and developed during regular research team meetings between authors 1, 6 and 7 and were triangulated with the other authors. Descriptions of the themes, subthemes and codes were developed and captured in a coding framework in NVivo 12 software. | 8-30 |
| 26. | Derivation of themes | Themes and subthemes were derived from the data using iterative inductive processes. | 8 |
| 27. | Software | NVivo 12 | 8 |
| 28. | Participant checking | Participant checking did not take place. Instead the lived experience researcher was involved in the theme identification process to enhance validity of the interpretation. Further, outside of the lay summary of the findings being returned, there was no formal opportunity for participants to feed back on the findings and recommendations other than contacting the researchers directly. | 7-8 |
| 29. | Quotations presented | Illustrative quotes from participants were used. Quotations are identified with participant type and number to ensure anonymity. Specifically, ^P1-27^ = young person and ^C1-12^ = carer. | 9-30 |
| 30. | Data and findings consistent | Data and findings are consistent throughout the manuscript. Young People and carers provided perspectives on EIPS support and there were many thematic similarities in their data. Accordingly, findings are presented together. Differences in participants’ (YP and carers) experiences accessing state and federally funded EIPS were highlighted where appropriate. | 9-30 |
| 31. | Clarity of major themes | Four key themes were identified as influential in shaping participant’s hospitalisation experiences: 1. A two-way street: EIPS affected how participants experienced hospitalisation, and vice versa; 2. It’s about people: the quality and continuity of relationships participants had with staff, in hospital and at their EIPS, was central to their experience; 3. A gradual feeling of agency: participants viewed EIPS as both reducing involuntary care and supporting their self-management; and 4. Care coordination as a GPS for the healthcare system: great when it works; frustrating when it breaks down. | 9-30 |
| 32. | Clarity of minor themes | Subthemes were clear and consistent throughout. Minor themes were discussed beyond subthemes in the context of the 4 major themes we identified; including a the importance of trusting relationship with a continuous care coordinator; a trauma-informed approach to care positively impacting hospitalization experiences; and conversely lack of coordination and a non-tailored approach leading to aversive experiences. | 9-30 |
