# Supplementary material for: The impact of early intervention psychosis services on hospitalisation experiences: a qualitative study with young people and their carers
Source: BMC Psychiatry. 2024 May 10;24:350. doi: 10.1186/s12888-024-05758-4 (PMC11088060; doi:10.1186/s12888-024-05758-4)
Supplement: Supplementary file 3 — Supplementary Material 3: Support Person Interview Guide [file 12888_2024_5758_MOESM3_ESM.docx]

Supplementary File 3. Interview Guide for Support People

| **Concept** | **Topic** | **Possible Questions** |
| --- | --- | --- |
| *Core Topics* | | |
| Pathway to care (access and expectations) | 1. Family/carer experience of coming into the program | 1. *Can you please share how you found out about this program?* 2. *Can you please share your story about coming into the program?*  - *Researcher to confirm whether referral to service for young person was via hospital. If yes, what was that experience like for you/ the young person you were supporting? If not, can you tell me about the process of coming into the program? Prompt: How long did it take between first noticing the young person you care for having trouble and connecting with hYEPP/EIP?*  1. *What were you hoping to get out of the program? Prompt: What did you think you and/or the young person you care for needed from the program?* |
| Engagement and perceptions of program appropriateness | 1. Family/carer experience of the program | 1. *Can you please share a few examples of the types of problems or issues that the program has helped you manage? Prompt: What types of support have your received from the program?* 2. *What aspects of the support provided work well or not so well for you? Prompt: Are you able to access support when you need it?* |
| Functional Outcomes associated with the program | 1. Client views on the impact of the program on their functional outcomes | 1. *We are interested in hearing your thoughts on the impact of the hYEPP/EIP program on different areas the young person you support’s life. Can you think of anything that has changed for them since accessing the program?*  - *Prompt if requiring further examples: This might include areas of their life such as employment or education, their relationships or social life, their leisure activities, their daily living skills (like looking after themselves, managing finances, looking after their home)*  1. *For each outcome mentioned ask: Can you tell me what this change means for you/ them?*  - *Prompt: is it important or not? Why is that?*  1. *Given these changes, can you tell me how the young person you support continues to manage their mental health or any symptoms? Probe: self-management, personal understanding of situation/ diagnosis, support networks.* 2. *How was the hYEPP/EIP staff/service involved in these changes in their life?* 3. *Were any other services or people involved in these changes?* |
| Hospitalisation experiences | 1. Family/carer hospitalisation experience whilst involved in the program | 1. *Did the young person you support have any hospital admissions while receiving hYEPP/EIP care?*  - *Prompt: If yes explore… how many times were they in hospital? Was it a voluntary or an involuntary admission? What was the impact of this experience on your/ their engagement with services?*  1. *What was the process of the young person you support going into hospital, being in hospital and transitioning out of hospital like for you?* 2. *What was the support from the hYEPP/EIP staff like for you/ them during this time?*  - *Prompt: Can you tell me how you found the communication between you, the hospital staff and the hYEPP/EIP service?* - *Prompt: Can you tell me how you felt about your involvement in their care?* - *Prompt: What aspects of the young person you support’s care did you focus on with the hYEPP/EIP service at this time?*  1. *Overall, do you feel that hYEPP/EIP involvement was helpful or not helpful to you/ the young person you support during this time? Why is that?*  - *Prompt: Is there anything the hYEPP/EIP team did particularly poorly or particularly well?* - *Prompt: Can you think of anything else you would have liked from the hYEPP/EIP team at this time?*  1. *Did the young person you support have any hospitalisation experiences when you were not with the hYEPP/EIP program? If yes, how do these experiences compare?* 2. *Could you tell us whether you feel that your family being a part of the hYEPP/EIP program impacted the length of the young person you support’s hospital stay or their admission to hospital in anyway?* |
| *Specific Fidelity Topics*  *Note: these topics may already have been addressed by participants in response to Question B. If not, these questions may be asked.* | | |
| Fidelity | 1. Treatment (medication, CBT, family care) | 1. *Can you please share what treatments the young person you care for was offered or received? (What, When, How, Who) Prompt: How are treatment decisions made?* 2. *What aspects work well or not so well for you and the young person you care for?* |
|  | 1. Ongoing community care, mobile outreach and group programs | 1. *Can you please share the kinds of support you have received? (Who, What, When, Why) Prompts: Has the young person you care needed crisis support or care after hours? Has the young person you care for attended group programs?* 2. *What aspects of this community support work well or not so well for you and the young person you care for?* |
|  | 1. Family programs and family peer support | 1. *Have you or any other family members, carer or friends participated in any of the family programs or family peer support programs? (Who, What, When, Why)* 2. *Can you share how these programs were helpful to you or the young person you care for, or not?* |
|  | 1. Youth participation and peer support program | 1. *Has the young person you care for visited the youth participation and peer support programs? (Who, What, When, Why)* 2. *What aspects of these programs work well or not so well for you and the young person you care for?* |
